# The Indiana University Cognitive Health Outcomes Investigation of the Comparative Effectiveness of dementia screening (CHOICE) study: study protocol for a randomized controlled trial

**DOI:** 10.1186/1745-6215-15-209

**Published:** 2014-06-06

**Authors:** Nicole R Fowler, Amanda Harrawood, Amie Frame, Anthony J Perkins, Sujuan Gao, Christopher M Callahan, Greg A Sachs, Dustin D French, Malaz A Boustani

**Affiliations:** 1 Department of Medicine, University of Pittsburgh, 200 Meyran Avenue Suite 300, 15215 Pittsburgh, PA, USA; 2 Indiana University Center for Aging Research, 410 West 10th Street Suite 2000, 46202 Indianapolis, IN, USA; 3 Regenstrief Institute, Inc., 410 West 10th Street Suite 2000, 46202 Indianapolis, IN, USA; 4 Department of Biostatistics, Indiana University School of Medicine, 410 West 10th Street Suite 3000, 46202 Indianapolis, IN, USA; 5 Center for Healthcare Studies and Department of Ophthalmology Northwestern University Feinberg School of Medicine, 420 East Superior Street, 60611 Chicago, IL, USA

**Keywords:** Alzheimer’s disease, Dementia screening, Dementia, Primary care

## Abstract

**Background:**

Dementia affects over 4 million people in the US and is frequently unrecognized and underdiagnosed in primary care. Routine dementia screening in primary care is not recommended by the US Preventive Services Task Force due to lack of empirical data on the benefits and harms of screening. This trial seeks to fill this gap and contribute information about the benefits, harms, and costs of routine screening for dementia in primary care.

**Methods/Design:**

Single-blinded, parallel, randomized controlled clinical trial with 1:1 allocation. A total of 4,000 individuals aged ≥65 years without a diagnosis of dementia, cognitive impairment, or serious mental illness receiving care at primary care practices within two cities in Indiana. Subjects will be randomized to either i) screening for dementia using the Memory Impairment Screen Telephone version or ii) no screening for dementia. Subjects who screen positive for dementia will be referred to the local Aging Brain Care program that delivers an evidence-based collaborative care model for dementia and depression. Research assistants will administer the 15-item Health Utility Index, Patient Health Questionnaire, Generalized Anxiety Disorder Scale, and Medical Outcomes Study at baseline, 1, 6, and 12 months. Information about advanced care planning will be collected at baseline and 12 months. All enrollees’ medical records will be reviewed to collect data on health care utilization and costs.

**Discussion:**

We have two primary hypotheses; first, in comparison to non-screened subjects, those who are screened and referred to a dementia collaborative care program will have a higher health-related quality of life as measured by the Health Utility Index at 12 months post-screening. Second, in comparison to non-screened subjects, those who are screened and referred to a dementia collaborative care program will not have higher depression or anxiety at one month post-screening as measured by the Patient Health Questionnaire and Generalized Anxiety Disorder Scale scales. Our secondary hypothesis is that screened subjects will have an Incremental Cost-Effectiveness Ratio below the maximum acceptable threshold of $60,000 per quality adjusted life year saved at 12 months.

**Trial registration:**

Ongoing; registered on September 19, 2012. ClinicalTrials.gov Identifier: 2012 NCT01699503.

## Background

Dementia is a debilitating chronic brain syndrome that is estimated to affect 4.5 million people in the United States [1,2]. Currently, Medicare beneficiaries with dementia account for 34% of Medicare spending, even though they constitute only 13% of the beneficiaries aged 65 and older. By 2050, Medicare spending related to dementia will surpass $1 trillion [3]. Most patients with dementia are cared for in primary care settings with the majority of these cases being unrecognized [4,5]. Some argue that screening for dementia in primary care is the optimal strategy to increase dementia recognition and thus reduce dementia-related societal cost [5,6]. However, the United States Preventive Services Task Force did not find any randomized controlled trials of routine dementia screening and concluded that the evidence to systematically screen for dementia in primary care is insufficient [7,8]. Nevertheless, the Center for Medicare and Medicaid Services is currently covering the costs of an Annual Wellness visit for Medicare beneficiaries that includes the detection of cognitive impairment [9]. Hypothesized benefits for dementia screening include the opportunity to identify reversible causes of impairment, initiate early pharmacological and non-pharmacological interventions, and enhance patient and caregiver education and planning about the disease and its course. Hypothesized harms include inducing depression and anxiety once diagnosed and labeling patients who might suffer from stigmatization.

In order to fill the gaps in the literature regarding the benefits and harms of dementia screening, scientists at the Indiana University (IU) Center for Aging Research are conducting a randomized controlled dementia screening trial called the IU CHOICE trial. The CHOICE trial will contribute important information to patients, families, providers, and policy-makers about the harms and benefits of routine screening for dementia in primary care. If this trial is successful, it not only will address the appropriateness of routine screening for dementia, it also will provide a template for the successful implementation of screening programs, coupled with diagnosis and treatment programs, that are practical for a broad range of health care systems.

The outcomes of the CHOICE trial are the impact of routine screening in primary care, coupled with a collaborative dementia care program, on patients’ health-related quality of life, mood, and anxiety. In addition, the CHOICE trial has a secondary outcome of the cost effectiveness of dementia screening in primary care. We hypothesize that screened subjects, compared with non-screened subjects, will have a higher health-related quality of life as measured by the Health Utility Index (HUI) at 12 months post-screening; screened subjects will not have higher depression or anxiety at one month post-screening (as measured by the Patient Health Questionnaire (PHQ-9) and Generalized Anxiety Disorder (GAD-7) scales); and screened subjects will have an incremental cost-effectiveness ratio (ICER) below the maximum acceptable threshold of $60,000 per quality adjusted life year (QALY) saved at 12 months.

## Methods/Design

### Design

**Figure 1:**
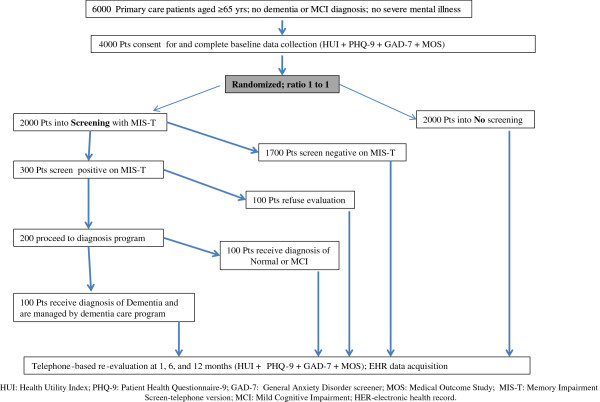
Study protocol flowchart.

This clinical study is a pragmatic, two-center, randomized, controlled, single-blind study of dementia screening in primary care, coupled with a collaborative dementia care program. The study will recruit 4,000 adults aged 65 and older who attend primary care clinics affiliated with Eskenazi Health (EH) and Indiana University Health (IUH) serving the cities of Indianapolis and Lafayette in Indiana. These subjects will be randomly assigned at a ratio of 1:1 into “no screening” or “screening” groups (2,000 patients per group; Figure 1). Subjects randomized into the screening group who have a positive screen will be further referred into an existing primary care-based collaborative dementia care program within the two health care systems. This collaborative dementia care program delivers evidence-based diagnostic assessment, counseling, and management for patients with dementia and their informal caregivers [10,11]. All patient outcomes are measured at baseline, 1, 6, and 12 months, in person or via telephone. Health care utilization and cost data for all subjects throughout the 12 month study period will be obtained from the Regional Health Information Exchange in the study recruitment area and the medical records of the local health care systems.

The study has been approved by the institutional review boards of Indiana University and Purdue University in Indianapolis, the University of Pittsburgh, and the local board of Indiana University Health at Arnett Hospital (IRB# 1206009010). The CHOICE trial is registered with clinicaltrials.gov, Clinical Trials.gov Identifier NCT01699503.

### Setting and study population

Subjects will be recruited through the Indiana University Practice-Based Research Network (IU-PBRN) and local electronic medical record (eMR) systems and will include 17 primary care centers affiliated with EH and IUH services [4]. These two systems serve a diverse population of insured and uninsured patients throughout the cities of Indianapolis and Lafayette, IN, USA, and represent more than 110 primary-care physicians, 100,000 patients, and 300,000 visits annually. Data generated within the IU-PBRN is collected and stored in the Indiana Network for Patient Care (INPC), which serves as the central Indiana Regional Health Information Exchange.

### Recruitment and screening

We anticipate the need to identify 6,000 older adults to enroll 4,000 subjects. Rolling enrollment will take place over 45 months with a monthly enrollment rate of approximately 90 subjects. Among the participants in the screening arm who screen positive, we anticipate that the local collaborative dementia care program will manage an estimated 100 subjects with dementia (Figure 1). The entire 4,000 subject cohort will be followed for 12 months.

The data managers of INPC or the local health care systems will extract a list of eligible patients based on the study’s inclusion criteria (e.g., patients cared for in the targeted primary care practice, over the age of 65, and without a diagnosis of dementia). The eligible list of patients will be provided to the primary care clinicians (PCPs) to authorize the study personnel to approach their patients for potential participation. Once approved by the PCPs, study personnel will approach eligible patients during their visit to their primary care clinic or via the telephone to confirm eligibility and obtain informed consent.

### Eligibility

Our target population are primary care patients aged 65 years or older without a diagnosis of dementia or any cognitive impairment in their medical record. Eligibility is established through a screening of the INPC database or the local practice medical record, and the assessments conducted by the research assistants face-to-face or via the telephone.

Inclusion criteria

• Adults aged 65 and older

• At least one office visit to their EH or IUH primary care physician within the previous year

• No previous diagnosis of dementia or cognitive impairment as determined by ICD-10 codes or the presence of prescription for anti-dementia medications (cholinesterase inhibitors or memantine)

• Ability to consent to participate in the study

• Ability to communicate in English

Exclusion criteria

• Adults who are a permanent resident of a nursing facility

• A serious mental illness such as bipolar disorder or schizophrenia as determined by the presence of related ICD-10 codes indicative of such an illness or

• A pre-existing diagnosis of dementia or cognitive impairment

Following the confirmation of eligibility and obtaining informed consent, all consenting subjects (screening and non-screening arms) will complete the baseline assessment which includes the HUI, the PHQ-9, the GAD-7, the Medical Outcome Study (MOS), and seven questions that inquire about the presence of an Advance Directive and Power of Attorney for health care and financial affairs. Following these assessments, if the participant has been randomized to the screening arm, the research assistant administers the Memory Impairment Screen (MIS-T). The MIS-T takes approximately four minutes to complete and has demonstrated excellent psychometric properties in primary care and community samples with a positive likelihood ratio of 33 [12,13]. The MIS-T has a total score from 0 point to 8 points. A cut-score <5 has 86% sensitivity and 91% specificity for dementia with a positive predictive value of 72% and negative predictive value of 96% in a setting with a dementia prevalence of 15% [14,15].

### Randomization and blinding

Randomization will occur at the patient level rather than at the level of providers or clinics to minimize the effects of unmeasured case mix differences and clinic-level clustering. We estimate that the risk for “spillover” from having participating clinics treat both intervention and usual care patients is likely to be small given the current low levels of detection of dementia in primary care [16,17]. If anything, patient-based randomization will conservatively bias the results in favor of usual care. Allocation of screening will be performed using a computer-generated system. Patients will be assigned to screening using stratified block randomization. Patients are randomized with a block size of 4 and stratified by health care system (EH or IUH).

At the beginning of the study, prior to enrollment, the study biostatistician, using SAS software version 9.3 (SAS Institute, Cary, NC, USA) generated a block randomization scheme to assign study identification numbers either a screen or no screen status. The CHOICE study coordinator, who is not blind to status, will prepare an envelope for each study identification number that includes a piece of paper indicating whether that study identification number has been randomized to the screen or no screen arm. The envelopes are sealed and then placed in recruitment packets which are delivered to the research assistants in sequential order. The research assistants open the randomization envelope at the time of enrollment, following administration of the baseline assessment, and administer the MIS-T if the participant has a number that was randomized to the screening arm. The participants and the study research coordinator who creates the randomization envelopes for the recruitment packets are not blinded to group assignment. However, the principal investigators of the study, the research assistants responsible for administering the outcome assessments, and the lead biostatistician all remain blinded to individual assignments. A second unblinded statistician is responsible for assessing all screening data and assisting with analyses that are reported to the Data Safety Monitoring Board (DSMB) for its review of protocol implementation. The DSMB, in turn, reports its findings to the project Principal Investigator and Co-Investigators. This ensures that all data about protocol deviations are available to the project leaders without revealing the identification of participants.

### Description of the intervention condition

Subjects who are randomized into the screening arm of the trial and score less than 5 points on the MIS-T (e.g., a positive screen) will be referred by the CHOICE study coordinator to the local collaborative dementia care program called the Aging Brain Center Medical Home (ABC MedHome). Subjects who screen positive on the MIS-T but are found to have no dementia by the subsequent assessment will be referred for an annual cognitive assessment with the local memory care practice at EH or IU Health.

The goal of the ABC MedHome program is to assist primary care clinicians in achieving the recommended standard of care in the diagnosis and management of older patients with dementia. Much of the intervention, facilitated by non-medical care coordinators, is targeted to co-manage or support the practice behavior of primary care clinicians, enhance self-management skills of both the care-recipient and the informal caregiver, and maximize the coping behavior of the patient and the informal caregiver. By design, the previously-tested program protocols lead to individualized and patient-centered profiles of actual interventions for individual patients and their informal caregivers [18,19]. The program has four main phases: the initial assessment phase, the plan of care development phase, the second home visit phase, and the follow-up phase.

In the initial assessment, the care coordinator conducts a demographic and medical information interview, reviews medication lists, and gathers and reviews any diagnostic testing and brain imaging results with the primary intention of identifying any potentially reversible causes of dementia and co-morbid conditions. After completing a pre-home-visit interview, the care coordinator travels to and conducts a face-to-face initial assessment at the patient’s and/or informal caregiver’s residence or preferred location. At the patient’s home, the care coordinator conducts a brief cognitive assessment, biopsychosocial needs assessment of the patient and informal caregiver, and medication reconciliation. The program uses standardized assessment tools including The Healthy Aging Brain Care Monitor (HABC-M) [10,11]. If there is no available or identified caregiver, the care coordinator attempts to identify a caregiver and complete the caregiver questions at a later date either by phone or in-person. The care coordinator documents the initial and follow-up visits using care coordination software called the eMR-ABC.

The second phase of the intervention is the development of an individualized Care Plan with an emphasis on coordinating care with the patient’s primary care provider. This phase begins after the first home visit and concludes with a second home visit by the care coordinator. After consultation and coordination with the ABC MedHome medical director (a geriatrician or a neurologist) and the patient’s primary care physician, the ABC MedHome care team (medical director and nurse) rules in or out the diagnosis of dementia and its subtypes and finalizes the individualized care plan with the primary care physician. If necessary, the patient will be referred for a more extensive cognitive and mental health evaluation at the local memory care practice.

Following the development of the care plan, the care coordinator will schedule a second face-to-face home visit with the patient and the informal caregiver within 2 to 4 weeks of the initial home visit. The key purpose of the second home visit is for the care coordinator to disclose the diagnosis, the natural history, and the prognosis of dementia; implement appropriate care protocols; review, explain, and distribute the corresponding educational handouts for both the patient and the informal caregiver; and connect patients and informal caregivers to in-home services and community resources as needed.

The fourth and final phase of the ABC MedHome is the follow-up and ongoing management of the patient. This includes interaction with the patient or the caregiver via face-to-face home or clinic visits, phone contact, or contact by email, fax, or mail. The minimum amount of contact during this time will be once monthly for the first three months and once every three months thereafter. During these interactions, the care coordinator will answer any questions generated from previous visits, collect patient and informal caregiver feedback, reconcile medications and review medication adherence, have the informal caregiver complete the HABC-M to trigger the use of specific care protocols, and facilitate the informal caregiver’s participation in a local Support Group Program sponsored by the local chapter of the Alzheimer’s Association. Throughout the duration of the follow-up phase, the team will continue to work with the patient, the informal caregiver, and the patient’s primary care provider to monitor, implement, and adjust as necessary the individualized care plan. The program care, services, and protocols during this phase are:

i) Self-management/caregiver skills enhancement

ii) Support group participation

iii) Informal telephone support

iv) Problem solving training

v) Reducing anticholinergic medications

vi) Prescribing FDA-approved medications

vii) Managing high vascular burden

viii) Monitoring and supporting of caregiver’s emotional and physical health

ix) Managing transitional care

x) Managing acute care problems

xi) Root-cause analysis of re-hospitalization or re-emergency room visits

xii) Care prioritization

xiii) Discharge criteria

### Description of control condition

The 2,000 subjects who are randomized into the no screening group will serve as a usual care control group and continue to receive their usual primary care, including a referral to the local memory care practices if their primary care provider suspects the presence of a cognitive problem at any time during the study.

### Primary and secondary outcome measures

The primary outcome measure will be patient health-related quality of life (HRQOL) measured at 1, 6, and 12 months among the entire 4,000 enrollees. We will use the 15-item HUI to determine the subject’s HRQOL and change in HRQOL [20]. The HUI is a generic, utility-based HRQOL instrument applied in patients with a wide range of medical conditions including dementia [21,22]. It has eight attributes: vision, hearing, speech, ambulation, dexterity, emotion, cognition, and pain. The individual health domain scores range from 0.00 (maximum impairment) to 1.00 (no impairment) and the multi-attribute (HUI index) scores, a multiplicative function of individual attribute levels, range from 0.36 to 1.00 with anchors 0.00 = dead and 1.00 = perfect health. Naglie et al. found that test-retest reliability exceeded the standard for adequate reliability of 0.70 in those with mild dementia (ICC = 0.75) [22]. Vickrey et al. [23] found that the mean HUI multi-attribute utility score for 408 primary care patients with dementia was 0.54 (SD = 0.23) and the mean one year change was -0.30 among 240 dementia patients who remained in their own home after one year of receiving the dementia care program whereas the mean one year change was -0.76 among 29 dementia patients who moved into a skilled nursing home facility.

The second primary outcomes for the CHOICE trial are patient mood and anxiety as measured by the PHQ-9 [24,25] and GAD-7 [26,27]. The PHQ-9 is a 9-item depression scale with a total score from 0 to 27, and the GAD-7 is a 7-item anxiety scale with a total score from 0 to 21. Both of these scales are derived from the Patient Health Questionnaire and have good internal consistency and test-retest reliability as well as convergent, construct, criterion, procedural, and factorial validity for the diagnosis of major depression and general anxiety disorders [24-27]. In our previous primary care studies of community-dwelling older adults, the mean PHQ-9 scores ranged from 3.8 (SD = 5.1) to 4.4 (SD = 5.6) and the mean GAD scores ranged from 2.7 (SD = 3.2) to 3.2 (SD = 3.5) [4,10,11]. The research assistants will notify the study coordinator if any subjects express thoughts or demonstrate tendencies of self-harm with a positive response on the PHQ-9 question about suicidal thoughts. The CHOICE study coordinator will keep a detailed log of these events and will notify the subject’s primary care provider immediately.

To conduct a cost effective analysis of CHOICE, we will use both the INPC database and the local eMR to identify all episodes of ambulatory or acute care utilization occurring within the 12 months before and after enrollment. We will structure continuous variables that describe the number of ambulatory and/or acute care episodes.

Additional outcomes of the trial include measures of social support, advance care planning information (e.g., having power of attorney for health care and/or financial affairs, having a living will, and having life and additional insurance policies), and evidence of any dementia recognition by providers using the same ICD codes used to identify eligible subjects. Additional data that will be collected includes patient age, gender, race, education, income, living situation, marital status, medications, and presence of any of 10 chronic conditions as indicated by ICD-10 codes in the medical record (arthritis, congestive heart failure, coronary artery disease, cancer, chronic obstructive pulmonary disease, diabetes, stroke, hypertension, kidney disease, and liver disease). Given the pragmatic design of CHOICE and that participants in the control group may receive dementia screening and dementia care processes as part of their routine primary care, the study will use the local eMR and the eMR-ABC MedHome to measure dementia care processes for both the control and the intervention groups. This includes all patient contacts by the local memory care or primary care practices, referrals to local Alzheimer’s disease support groups, the use of home health services, and assessment of the level of participation of patients and caregivers in the ABC MedHome program. An overview of the data is presented in Table 1.

**Table 1:** Overview of data sources

**Construct**	**Source of data**	**Specific variables/instruments**
Social, demographic, advance care planning	Study survey	MOS, age, gender, race, education, income, living situation, marital status, and advance care planning
Health related quality of life, mood and anxiety	Study survey	HUI, PHQ-9, GAD-7
Health care utilization	Indiana Network for Patient Care records or the local eMR at IUH Arnett for the 4,000 subjects	ER episode, location
		Hospital episode, location
		Inpatient diagnoses (ICD-10 codes)
		Length of stay
Co-morbidity	Indiana Network for Patient Care records or the local eMR at IUH Arnett for the 4,000 subjects	ICD-10 codes for the 10 common chronic diseases
Medication use	Indiana Network for Patient Care records or the local eMR at IUH Arnett for the 4,000 subjects	Psychotropics
Care Processes	eMR-ABC and INPC	Visits to memory care practice.
		Collaborative dementia care team visit.
		Visits with HABC-M
		Protocols delivered by the care team

### Analysis plan

#### Study arm comparison

To verify the comparability of the randomized groups, baseline characteristics of the screened and no screening patient groups will be compared using *t*-tests for continuous variables and *χ*^2^ tests for categorical variables. We will carefully examine the distributions of continuous variables and use alternative approaches such as transformation or non-parametric methods in cases of violation to the normal distribution assumption. We also will examine the frequency distribution of all categorical variables and adopt exact inference procedures in cases of zero or small cell size. We will compare the baseline characteristics of subjects with missing outcomes due to death or refusal at 12 months to subjects who complete the follow-up to detect potential violation to the missing at random assumption. Further sensitivity analyses will be performed using various methods of imputation or a full parametric likelihood approach assuming various patterns of missing data [28].

Analyses for the primary aim will compare mean measures of HRQOL from the HUI in the screening group to the no screening group using analysis of covariance (ANCOVA) while including baseline measures as covariates. We will first conduct the ANCOVA models separately for outcomes obtained at 12 months while adjusting for baseline measures. We will then use the mixed effect models to examine whether the difference between the two groups changes over time using repeated HUI measures as the dependent variables and group, time, and interaction between groups as independent variables while controlling for other baseline covariates. Patients who do not complete the 12-month surveys for HRQOL will be excluded from the analyses followed by sensitivity analyses on missing data.

Analyses for the secondary outcome measures will compare change in depression and anxiety levels post dementia screening between screened subjects and subjects in the no screening group. ANCOVA models will be used to compare change in PHQ-9 and GAD-7 scores from baseline to 1 month between the screening and no screening groups adjusting for patients’ characteristics and baseline measures. Mixed effect models similar to those used for HUI will be used to determine whether depression and anxiety changes over time are different for the two groups.

For the cost-effectiveness aim, we will estimate the cost of the dementia screening program in comparison to no screening. The estimations will include the cost of the collaborative dementia care program for those who screen positive. This economic evaluation will be conducted from both the societal and payer (Medicare) perspectives. The societal perspective will capture caregiver and patient time and transportation costs and the effect on quality of life for patients and caregivers [29]. Medicare payments for health care utilization will be valued. Costs will be divided into fixed and variable costs and by the screening, initial assessment phase, and follow-up phase of treatment. Total medical care utilization costs will be estimated for the control and screening groups from health care utilization and reimbursement data captured by both INPC database and the local medical records of EH and IUH during the trial. The cost analysis will control for non-dementia related conditions such as stroke, cancer, and diabetes that are known to be associated with increased health care utilization and costs. Program or activity costing will be conducted to estimate the costs necessary to implement and operate the screening and collaborative dementia care management program and the usual care for dementia patients. The program costs can be compared to the risk adjusted economic savings estimate comparing the intervention to usual care for the calculation of net present value and cost benefit ratios. Patient and caregiver time, staff time, fringe benefits, overhead costs, and materials will be logged by staff using the care coordination software used by the ABC MedHome and assessed for the 12-month follow-up period. Transportation costs will be estimated from data on distance from home to clinic. Health care contacts will be dated and checked to avoid double counting via claims and activity costing methods. Prescription drugs will be valued at the median wholesale price, and a dispensing fee will be added for each 100 doses. Unit wage costs and fringe benefits will be standardized applying median values paid to personnel in the region, with wage data collected from the health care system partners. Costs will be accumulated during the trial based on the probability of survival each month times the monthly cost of care for survivors to address censoring [30]. Effects and costs will be discounted at a 3% annual rate for the trial period. The ICER, will compare the no screening and less resource intensive usual care treatment strategy (UC) with the screening coupled with the more intensive treatment intervention (the collaborative dementia care program) using the following formulas for (1) dementia patients (AP) and (2) Dementia patients plus their caregivers (CG).

(1) $$\begin{array}{l} \text{ICE}R_{\mathrm{AP}}=\left[\text{COS}T_{\text{HABC}}-\text{COS}T_{\mathrm{UC}}\right]\\ \;÷\left[\text{QAL}Y_{\text{HABC}}-\text{QAL}Y_{\text{UC}}\right] \end{array}$$

(2) $$\begin{array}{l} \text{ICE}R_{\mathrm{AP}+\mathrm{CG}}=\left[\text{COS}T_{\text{HABC}}-\mathrm{COS}T_{\mathrm{UC}}\right]\\ \;÷\left[\text{QAL}Y_{\text{HABC}}-\text{QAL}Y_{\mathrm{UC}}\right] \end{array}$$

Uncertainty in the cost-effectiveness ratios and 95% confidence intervals will be assessed with 1,000 bootstrap samples. The outcome of each sample will be expressed as a scatter plot of incremental costs and effects generated from the bootstrap samples, reflecting the uncertainty arising from the model parameters. Results will also be displayed using net benefit analysis and cost-effectiveness acceptability curves (CEAC) [31]. There is controversy about the appropriate ceiling ratio for health benefits [32]. Garber et al. have recommended that a value of twice the median annual per capita income will result in efficient resource allocation [32].

The effects on ICERs of alternative unit cost estimates for caregiver time value and overhead cost rate for the screening and coordinated care management will be examined in a series of one-way and multi-way sensitivity analyses. The ceiling ratio λ will be varied from $30,000 to $100,000 per QALY for the CEAC analysis. The cost-effectiveness hypothesis will be tested against the $60,000 per QALY maximum willingness to pay norm [33].

#### Justification of sample size

Assuming 85% sensitivity of the MIS-T screening instrument and 15% prevalence of dementia in this patient population, to achieve 80% power to detect a significance effect size of 0.094 between the screening group and the no screening group at α = 0.05 level (two-sided), and allowing 10% of patients with missing follow-up outcomes at 12 months, we need to enroll at least 3,951 patients into the study. The effect size of 0.094 for the screening group and the no screening group reflects a difference of 0.40 standard deviation (SD) between demented patients in the collaborative dementia care program and demented patients who were not screened and a difference of 0.06 SD in the majority of patients who are not demented in either group. We demonstrate, in Table 2, that our planned sample size of 4,000 will have sufficient power to detect significant differences between the screen group and the no screen group under various scenarios assuming varying degrees of efficacy measures for the collaborative dementia care program and for the screening only subjects.

**Table 2:** **Estimated power with a total sample size of 4,000 comparing primary outcome measures between the screening group and the no screening group (two-sided at α = 0.05) allowing 10**% **of patients with missing outcomes at 12 months**

**Effect size of collaborative care in demented subjects**	**Effect size in non-demented subjects**	**Observable effect size**	**Power estimate**
0.3	0.07	0.091	77.9
	0.08	0.100	85.1
0.4	0.07	0.102	86.4
	0.08	0.110	91.0

For the second primary aims of change in patient mood and anxiety, given the sample size of 4,000, we will have greater than 95% power to test the equivalence levels in PHQ-9 and GAD-7 at 1 month assuming equivalence differences of 0.6 (SD = 5.1) on PHQ-9 and 0.5 (SD = 3.2) on GAD-7 based on our previous studies of primary care patients [34-38].

## Discussion

Despite the availability of pharmaceutical and life-style interventions that show some benefit in the treatment of dementia [7,8,39-41], there is no cure. Results from the literature indicate that most older adults would accept screening for dementia [37,42], yet few primary care physicians conduct dementia screening, and as many as 50% of PCPs are unaware of their older patients’ cognitive status [43,44]. While the United States Preventive Services Task Force has determined that the need for screening non-symptomatic patients for dementia in primary care is unsubstantiated, the Center for Medicare and Medicaid Services and expert recommendations on the comprehensive care of older adults encourage early detection of dementia [6,9,4].

The CHOICE Trial is the first study to assess the harms, benefits, and cost effectiveness of screening for dementia. CHOICE will compare patient outcomes of screening when linked to a state-of-the-art treatment program and no screening among 4,000 older adults cared for in typical urban and suburban primary care practices. In this pragmatic trial, patients in the no screening control arm may be screened as part of routine primary care. Patients in the screening arm who screen positive will be referred to an evidence-based best practice model, the ABC MedHome, for assessment and long-term management of the patient and caregiver.

The CHOICE trial has some limitations. The major limitation is its short 12-month follow-up duration. Dementia is a chronic condition that worsens over time; while one year of observation will provide time to monitor changes in the outcomes selected for this trial, it does not provide a long enough observation window for more distal outcomes such as nursing home placement or mortality. From prior studies, we know that one year of follow-up data is sufficient to detect the impact of the collaborative dementia care program on screened detected patients with dementia. In our clinical trial of the collaborative dementia care program, we were able to identify a clinically relevant effect within 12 months [10,11,18,19].

Perhaps the largest threat to the study as proposed, is meeting the enrollment target. The research infrastructures available for our study and the extensive experience of our investigators in enrolling similar large and vulnerable populations greatly increase our likelihood of success in recruiting 4,000 subjects within 45 months. In addition, we have contingency plans to double our number of potentially eligible subjects by using additional IU-affiliated primary care clinics with access to more than 30,000 older adults aged 65 and older.

## Trial status

Ongoing and as of December 31, 2013, the trial has recruited more than 750 subjects.

## Abbreviations

ABC MedHome: Aging Brain Center Medical Home; ANCOVA: Analysis of covariance; CEAC: Cost-effectiveness acceptability curves; DSMB: Data Safety Monitoring Board; EH: Eskenazi Health; eMR: Electronic medical record; GAD-7: Generalized Anxiety Disorder Scale; HABC-M: Healthy Aging Brain Care Monitor; HUI: Health Utility Index; HRQOL: Health-related quality of life; ICD-10: International Classification of Diseases, 10th Revision; ICER: Incremental Cost-Effectiveness Ratio; INPC: Indiana Network for Patient Care; IU: Indiana University; IU CHOICE: Indiana University: Cognitive Health Outcomes Investigation of the Comparative Effectiveness of Dementia Screening Study; IUH: Indiana University Health; IU-PBRN: Indiana University Practice-Based Research Network; MIS-T: Memory Impairment Screen; MOS: Medical Outcome Study; PCP: Primary care clinicians; PHQ-9: Patient Health Questionnaire; QALY: Quality adjusted life years.

## Competing interests

The authors declare that they have no competing interests relevant to the present manuscript.

## Authors’ contributions

NF: Manuscript writing, critical revision, and final approval of the manuscript. AH: Manuscript writing and final approval of the manuscript. AF: Study coordination, conception and design, manuscript writing, and final approval of the manuscript. AP: Planning of the statistical analysis and the sample size calculation, manuscript writing, and final approval of the manuscript. SG: Conception and design, planning of the statistical analysis and the sample size calculation, manuscript writing, final approval of the manuscript. CC: Conception and design, manuscript writing, critical revision, and final approval of the manuscript. GS: Conception and design, manuscript writing, critical revision, and final approval of the manuscript. DF: Planning of the statistical analysis, manuscript writing, and final approval of the manuscript. MB: Conception and design, manuscript writing, critical revision, and final approval of the manuscript. All authors read and approved the final manuscript.
